# Bioengineering facets of the tumor microenvironment in 3D tumor models: insights into cellular, biophysical and biochemical interactions

**DOI:** 10.1002/2211-5463.70018

**Published:** 2025-03-27

**Authors:** Salma T. Rafik, Deniz Bakkalci, Alexander J. MacRobert, Umber Cheema

**Affiliations:** ^1^ UCL Centre for 3D Models of Health and Disease, UCL Division of Surgery and Interventional Science, Faculty of Medical Sciences, Charles Bell House University College London UK; ^2^ Department of Clinical Pharmacology, Faculty of Medicine Alexandria University Egypt

**Keywords:** 3D models, cancer, tissue engineering, tumor microenvironment, tumor‐stroma interaction

## Abstract

The hallmarks of cancer extend beyond genetic anomalies to encompass a sophisticated tumor microenvironment, involving interactions between cancer and non‐cancer cells within a dynamic biophysical setting, influencing cancer progression. The tumor microenvironment is multifaceted, and it is increasingly clear that the interaction and interdependence of these different facets need to be better understood. Tissue engineering of 3D *in vitro* models of the tumor microenvironment provides an opportunity to study these interactions and their interdependence on cancer progression. Cancer metastasis still poses a major challenge, accounting for 90% of cancer‐related deaths. This accentuates the critical need to establish patient‐specific model systems that replicate tumor complexity at all stages of progression. Herein, we outline the latest advancements of *in vitro* 3D models of the tumor microenvironment and the different tools utilized to analyze such models. Henceforth, the interaction of the multifaceted tumor microenvironment can be elucidated using such sophisticated *in vitro* tools.

## Graphical abstract

The tumor microenvironment is a dynamic, multifaceted complex system of interdependent cellular, biochemical, and biophysical components. Three‐dimensional *in vitro* models of the tumor microenvironment enable a better understanding of these interactions and their impact on cancer progression and therapeutic resistance.

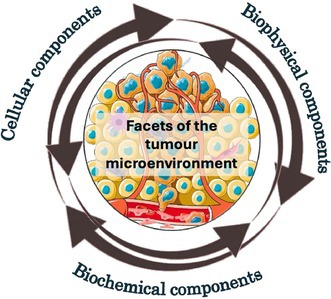

**Abbreviations**
ABC: transporter ATP‐binding cassette transporter
ARG/IDO: arginase/indoleamine 2,3‐dioxygenase
CAFs: cancer‐associated fibroblasts
CD: cluster of differentiation
CRC: colorectal cancer
CSF: colony‐stimulating factor
CTLs: cytotoxic T lymphocytes
CXCL, CCL: chemokine ligand
CXCR: chemokine receptor
DKK‐1: Dickkopf‐1
EC: endothelial cell
ECM: extracellular matrix
EMT: epithelial‐to‐mesenchymal transition
FAP: fibroblast activation protein
FGF: fibroblast growth factor
FN1: fibronectin‐1
FSP‐1: fibroblast‐specific protein 1
HGF: hepatocyte growth factor
HIFs: hypoxia‐inducible factors
IFN: interferon
IFP: interstitial fluid pressure
IGFBP: insulin‐like growth factor binding protein
IL: interleukin
kPa: kilopascal
MDSCs: myeloid‐derived suppressor cells
MIF: macrophage migration inhibitory factor
MMPs: matrix metalloproteinases
MSCs: mesenchymal stem/stromal cells
PAK1: P21‐activated kinase 1
PDAC: pancreatic ductal adenocarcinoma
PDGFR: platelet‐derived growth factor receptor
PD‐L1: programmed death ligand 1
PI3K‐AKT‐SELE/VCAM1: phosphatidylinositol 3‐kinases (PI3K)/protein kinase B/E‐selectin, vascular cell adhesion molecule‐1
PSCs: pancreatic stellate cells
ROS: reactive oxygen species
SDF‐1: stromal cell‐derived factor 1
TAMs: tumor‐associated macrophage
TGF‐β: transforming growth factor‐β
TILs: tumor infiltrating lymphocytes
TLR4: toll‐like receptor 4
TME: tumor microenvironment
TNF: tumor necrosis factor
TRAIL: tumor necrosis factor‐related apoptosis‐inducing ligand
Treg: regulatory T cells
VEGF: vascular endothelial growth factor
YAP/Taz: yes‐associated protein/Transcriptional coactivator with PDZ‐binding motif
α‐SMA: α‐smooth muscle actin

Despite significant therapeutic advances, certain tumor types, including glioblastoma, pancreatic cancer, and triple‐negative breast cancer, show limited treatment success [1]. Moreover, the development and screening of new anticancer drugs is a protracted and inefficient process, with <4% of drug candidates progressing through clinical trials and gaining FDA approval [2]. The lack of preclinical models that accurately mimic tumor complexity is one of the main causes of the disparity between preclinical research findings and clinical trial outcomes. Traditional *in vitro* 2D cell cultures in cancer research provide rapid but limited results due to the lack of biomimicry and physiological accuracy for drug discovery [3]. Cell culture in a 2D configuration alters cell morphology, cytoskeletal and nuclear morphology, affecting gene and protein expression [4]. Additionally, the lack of proper spatial cell–cell and cell‐extracellular matrix interactions in native tissues disrupts signaling pathways, causing adverse effects on cell viability, proliferation, and differentiation, and potentially the accumulation of genetic mutations [4]. 3D cell culture models can effectively mimic tumor architecture, behavior, histopathological features, genetic signatures, molecular profiles, and drug responsiveness seen *in vivo*, enabling the study of tumor cells and their microenvironment [5]. These models accurately capture biophysical and biochemical signals that drive cancer development, including tissue stiffness and the presence of oxygen/nutrient gradients [6]. In this review, we outline the different facets of the tumor microenvironment and their roles and interdependence in tumor progression. We present an overview of 3D *in vitro* models depicting tumor‐stroma interactions and their impact on the development of drug resistance.

## Tumor microenvironment (TME)

**Fig. 1:**
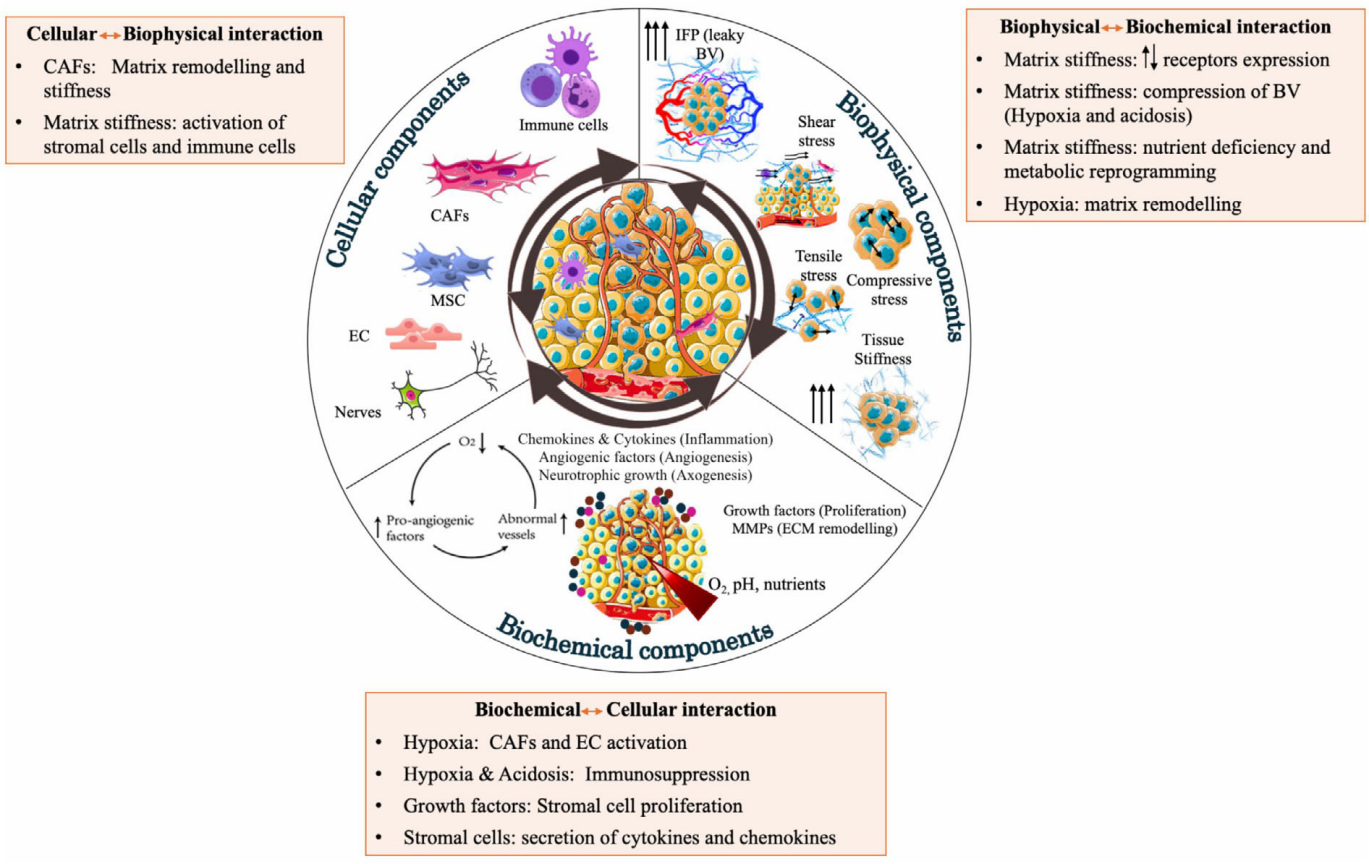
Schematic of the key component dictating the TME. Cellular components; cancer cells and stromal cells (fibroblasts, immune cells, mesenchymal stem cells, endothelial cells). Non‐cellular components include biophysical components: mechanical forces, tissue stiffness and geometry. The biophysical components dictate the formation of gradients of nutrients, oxygen and pH across different regions, which impact biochemical components including growth factors, enzymes, chemokines. Schematic generated using Servier Medical Art. TME, tumor microenvironment.

Tumorigenesis is an intricate process involving genetic, epigenetic, and metabolic changes and interactions with the microenvironment that transform normal cells into malignant ones [7]. Research shows tumors are not simply cancer cell accumulations but complex, dynamic microenvironments exposed to various physical and chemical stimuli, impacting cancer progression and drug resistance. Tumors include cancer and non‐cancerous cells, extracellular matrix (ECM), and soluble factors [8]. The tumor microenvironment (TME) varies across cancer types. The TME can be regarded as a sophisticated network of interdependent reciprocal communications where each component of the TME significantly influences the actions of others (Fig. 1). In solid tumors, non‐cancerous cells, such as fibroblasts, endothelial cells, and immune cells, can be recruited either locally or systemically (Table 1). Interactions within the TME can occur directly through cell‐to‐cell contact facilitated by adhesion molecules, electrical coupling, and the transit of signals through gap junctions or indirectly via classical paracrine signaling mechanisms, including cytokines, growth factors, and extracellular vesicles, as well as metabolite‐mediated communication [9].

**Table 1:** Various types of cells in the TME and their role in tumor progression.

	Cell type	Markers	Mechanism of interaction	Role in tumor progression	Possible targeting for cancer treatment
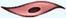	Cancer‐associated fibroblasts (CAFs)	α‐SMA, (FAP), (FSP‐1), and (PDGFR‐α and ‐β)	Secretion of soluble factors (SDF‐1, HGF, FGF, TGF‐β, VEGF, IL‐6) ECM remodeling (secretion of collagen type I, MMPs) Induction of epigenetic alterations of tumor cells Modulating tumor cell metabolism	Pro‐tumourigenic	Depletion of CAFs (anti‐FAP antibodies) Reprogramming of CAFs to quiescent state (all‐trans‐retinoic acid, CXCR4 inhibitor) CAFs activation blocking
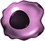	Tumor‐associated macrophage (TAMs)	CD68, CD86, CD11c (M1 phenotype) CD163, CD204, CD206 (M2 phenotype)	M1 TAM secrete IL‐1β, IL‐6, IL‐12, IL‐23, CXCL9, and CXCL10. M2 TAM secrete IL‐10, TNF, VEGF, PDGF, CSF‐1, MMPs, CCL17, CCL18, CCL22, and CCL24 (induction of angiogenesis, tumor invasion, suppression of T cells, ECM remodeling)	M1 TAM (pro‐inflammatory, anti‐tumourigenic) M2 TAM (anti‐inflammatory, Pro‐tumourigenic)	M2 to M1 reprogramming (CD47 Inhibitors, CD40 mAb Agonists) Depletion of M2 TAMs (CSF‐1R Inhibitors) Blocking recruitment of TAMs to cancer cell (CCR2 inhibitors and anti‐CCL2 antibodies)
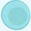	Tumor infiltrating lymphocytes (TILs)	CD8 cytotoxic T lymphocytes (CTLs), CD4 T‐ Helper, Treg (CD3, CD4, CD25, CD127, FoxP3)	Cytotoxic T secrete IFN‐γ, IL‐2, TNF‐α, Granzyme B, Perforin. CD4 T‐ Helper 1 & 17 (IFN‐γ, IL‐2, TNF‐α, IL‐21, IL‐17a, IL‐17f) Treg secrete IL‐10, TGF‐β, IDO, IL‐35	Cytotoxic T (anti‐tumourigenic) CD4 T‐Helper 1 & 17 (anti‐tumourigenic) Treg (pro‐tumourigenic)	Immune checkpoint (CTLA4, PD1, LAG3, B7‐H3, TIM3, VISTA) inhibitors Adoptive T cell transfer therapy Targeting Co‐stimulatory molecules and cytokine receptors on Treg cells (e.g. anti‐ CD25 antibody)
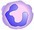	Myeloid‐derived suppressor cells (MDSCs)	CD11b + CD33 + HLA‐DR−/low	Produce arginine, NO, ROS, IL‐10 that inhibit activity of T cells and natural killer cells, promote angiogenesis and metastasis	(pro‐tumourigenic)	Depletion of MDSCs (CD33‐targeted antibody–drug conjugate) Differentiation of MDSCs (Toll‐like receptor 9 agonist) Inhibition of MDSCs immunosuppressive activity (ARG/IDO inhibitors)
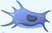	Mesenchymal stem cells (MSCs)	CD73, CD90, CD105	MSC1: Secretion of DKK‐1, IFNα, TRAIL, IGFBP (inhibition of angiogenesis, cell cycle arrest) MSC2: Secretion of CCL5, TGF‐β, IL‐6, VEGF, IL‐8, IL‐10, MMPs (Induction of angiogenesis, metastasis, enhanced tumor stemness, immunosuppression, differentiation into CAF).	Early stages (anti‐tumourigenic), later (pro‐tumourigenic)	MSCs derived extracellular vesicles (reduce angiogenesis, EMT and enhance cancer cell apoptosis) Genetically modified MSC (expressing IFN‐γ, IL‐2, TRAIL) TLR4 agonists reprograms into pro‐inflammatory MSC1
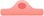	Endothelial cells	CD31, CD34, CD61	Produce angiocrine factors endothelin‐1, PDGF, bFGF, TGF‐β, IL‐6, IL‐8	Pro‐tumourigenic	Anti‐angiogenic drugs.
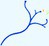	Nerves	Adrenergic receptors, acetylcholine receptors, neurotrophin receptors, NGFR	Release neurotrophins, IL6, VEGF promoting cancer cell proliferation, stemness, invasion, infiltration of Treg and MDSC and polarization of M1 to M2	Pro‐tumourigenic	β‐adrenergic receptors antagonists

## Biophysical aspects of the TME

### Tumor tissue architecture

The architecture of tissues is fundamentally linked to the maintenance of tensional homeostasis, which is crucial for organ functionality [10]. This architecture is shaped by the integrity and properties of both cellular and extracellular components, which depend on effective adhesive interactions between cells and the ECM. As the tumor expands, the organized structure of tissues is disrupted. The 3D architecture of the tumor tissue is often characterized by irregularity, heterogeneity, and disorganization, which can alter the mechanical microenvironment. The diverse architectural features of tissues, including their geometry, confinement, and fluid dynamics, impose varying mechanical stimuli on cancer cells located within the tumor. These mechanical stimuli significantly influence the behavior of cancer cells and their invasive potential [11].

### Role of ECM and tissue stiffness

The ECM plays a crucial role in transforming the physiological microenvironment into a tumor‐promoting microenvironment, regulating cancer cell proliferation, stemness, epithelial‐to‐mesenchymal transition (EMT), metastasis, angiogenesis, and therapeutic resistance [12]. The ECM undergoes continuous remodeling during tumorigenesis through the synthesis and degradation of various proteins [13]. Diverse stromal cell types and tumor cells can produce ECM proteins; nevertheless, CAFs serve as the primary source for the secretion, assembly, and modification of the ECM (Table 1). Excessive deposition and crosslinking of ECM proteins lead to the formation of dense ECM, thus increasing stroma stiffness [14]. Breast cancer tissue has a stiffness ranging from 4 to 12 kPa, compared to normal breast tissues at 0.4–2 kPa [15]. Tissues, including lung, brain, bone, and liver, have higher stiffness levels where cancer is present [15]. ECM stiffness, spatial distribution, and collagen fiber characteristics such as local densification, fibril straightening/stretching, and alignment near the boundary of the growing tumor significantly impact tumor progression and patient survival [16]. This occurs mainly through the promotion of proliferation [17], deformations of adjacent cells [18], increased cell traction forces [19], altered gene expression [20], loss of cell polarity, and increased metastatic potential of cancer cells [21]. ECM stiffness also impacts stromal cell behavior by triggering stromal fibroblast differentiation and macrophage polarization towards the pro‐inflammatory phenotype. The stiff ECM acts as a physical barrier, inhibiting immune cell infiltration and drug delivery [22].

### Mechanical forces in tumors

Mechanical force, initiated by cell–cell or cell‐ECM interactions, is transmitted via mechanical signaling pathways in a process known as mechano‐transduction [23] playing a crucial role in regulating oncogenesis, tumor progression, and metastasis [24]. These forces, categorized into solid stress, tension, and shear stress, affect tumor advancement and aggressiveness, which depend on the magnitude, duration, and direction of the applied forces, as well as the material properties (viscoelasticity and stiffness) of the cellular and extracellular tissue components [25]. Solid stress is defined as the stress contained within the solid phase of tumors, which encompasses the pressure applied by adjacent healthy tissues that act to limit the tumor's expansion as it proliferates. This is caused by cancer cell growth, stromal cell recruitment, and extra dense ECM, with the solid tumor core experiencing higher stress than border areas. Tensile stress is the result of the push and pull forces exerted by cells on crosslinked ECM fibers. The solid stress found in the interior of a tumor is primarily compressive, which tends to shrink the volume of an object [26]. In contrast, at the interface between the tumor and surrounding healthy tissue, the stress becomes tensile, indicating a propensity to enlarge the size of an object. Fluid shear stress is the internal frictional force between moving layers in laminar flow. Cancer cells experience two primary forms of fluid shear stress: vascular blood movement and interstitial flows within the tumor microenvironment. As the tumor continues to grow, newly formed blood vessels emerge; however, these vessels are distorted, highly permeable, and leaky, leading to the infiltration of liquids and macromolecules into the interspace, giving rise to an elevation in both blood viscosity and interstitial fluid pressure [27]. Fluid shear stress is significantly involved in vascular remodeling and the regulation of tumor cell growth, metastasis, and transport [28].

## Biochemical aspects

Abnormalities in vasculature, lymphatics, and ECM remodeling disrupt mass transport homeostasis in the TME, leading to a distinctive spatiotemporal distribution of nutrients, oxygen, metabolites, chemokines, and growth factors. This disrupted mass transport in the TME manifests as hypoxia, acidosis, localized accumulation and trapping of different chemokines and growth factors in the tumor core due to high interstitial fluid pressure. Consequently, this leads to the promotion of tumor growth and metastasis.

### Impact of Hypoxia gradient and metabolic shift

Hypoxia arises in solid tumors due to the rapid growth of cancer cells outgrowing their blood supply, creating regions with low oxygen levels (hypoxic core) within the tumor mass. Hypoxia can significantly impact cancer cells and the surrounding microenvironment, which further promotes tumor progression. Hypoxia can result in the expansion of more aggressive subclones of tumor cells mediated by hypoxia‐inducible factors (HIFs) that support cell survival by inducing compensatory angiogenesis, ECM remodeling, metabolic reprogramming, and immune suppression [29]. Hypoxic cancer cells in the core generate excess lactate from upregulated glycolysis, which is then released into the extracellular space, causing lactate‐dependent reduction of pH in some parts of the TME, leading to the development of a pH gradient [29]. As a result of this acidic environment, increased expression and activation of pH‐sensitive metastasis‐promoting proteins such as MMP‐2 and MMP‐9 occur and significantly promote the invasive potential of tumor cells [30]. In cancer cells, hypoxia can impact the response to chemotherapy in two main ways [31]. Firstly, it can enhance the ability of tumor cells to withstand higher concentrations of chemotherapeutic agents via blocking apoptotic pathways that trigger cell death, such as the TRAIL pathway [32]. Secondly, hypoxia promotes DNA repair mechanisms in cancer cells, thereby reducing the efficacy of multiple chemotherapeutic agents that cause DNA damage [33]. Additionally, the acidic TME plays a role in diminishing the cellular uptake of weakly basic chemotherapeutic drugs resulting in their accumulation in the extracellular space outside of their target sites in a process known as ion trapping, consequently impairing their effectiveness [31].

### Impact of the interstitial fluid pressure gradient and signaling factors

An additional feature of altered mass transport in the TME is the elevation of interstitial fluid pressure leading to the generation of shear forces and mechanical strain on tumor cells. In turn, this enhances their invasive capabilities through factors such as flow direction (transmural vs. luminal) [34], flow strength [35], as well as through the redistribution of chemokine gradients [36]. Fluid flow not only affects tumor cells but also influences the behavior of stromal cells such as fibroblasts and macrophages, which can independently facilitate tumor cell invasion [12]. Chemokines and growth factors are integral to the metastatic process of tumor cells and tissue tropism. Tumor cells secrete chemoattractants that recruit a variety of immune (e.g. macrophages, neutrophils) and stromal cells (e.g., mesenchymal stem cells, fibroblasts), which in turn produce more chemokines and growth factors that further promote tumor cell migration. This dynamic cancer/stromal cellular interaction is supported by the selective expression of specific receptors for the secreted factors, creating a self‐reinforcing paracrine loop [37]. Tumor cells can generate local gradients that promote their directed migration. For instance, tumor‐associated macrophages secrete epidermal growth factor, which subsequently activates the epidermal growth factor receptor present on breast tumor cells, thereby promoting their migratory behavior [38]. Furthermore, the CXCL12/SDF‐1 chemokine produced by CAFs is instrumental in driving the metastasis of CXCR4‐expressing breast cancer cells specifically to the lymph nodes and lungs [39]. Table 2 below summarizes the differences between normal tissues and tumor tissues.

**Table 2:** Comparison between characteristics of normal tissues and tumor tissues.

	Normal	Tumors
Cellular level		
Shape	Uniform, Spheroid shape	Irregular (Varied sizes)
Nucleus	Single nucleus	Irregular shape, multi‐nucleation
Chromatin	Fine, evenly distributed	Coarse, aggregated
Nucleolus	Single, inconspicuous nucleolus	Multiple enlarged nucleoli
Cytoplasm	Large cytoplasmic volume	Small cytoplasmic volume
Growth	Controlled	Uncontrolled
Maturation	Mature into specialized cells	Remain undifferentiated
Cell function	Perform designated tasks	Fail to perform designated tasks
Cell–cell adhesivity	Active Cell–Cell junction and contact inhibition	Reduced cell–cell junction and loss of contact inhibition
Cell stiffness	Normal stiffness	Softer, increase cell deformability
Cell contractility & location	Cells stick together and remain in intended location	Cells more contractile, can spread to other sites
Oxygen	Favor aerobic respiration	Favor anaerobic respiration (thrive in hypoxic conditions)
Energy efficiency	Very high	Very low production of ATP
Immune system	Can be eliminated by immune system	Cells evade immune system
Cell repair	Damaged cells are repaired or replaced	Cells are neither repaired nor replaced
Tissue‐level Biophysical factors		
Architecture	Organized arrangement polarized epithelial layers, preserved basement membrane	Disorganized, loss of polarity of epithelial layers and break down of basement membrane
Tissue boundary	Tissues clearly demarcated	Poorly defined tumor boundaries
Stiffness	Normal	Increased stiffness and physical stress
ECM	Normal ECM turnover	Excess ECM deposition, remodeling
IFP	Most normal tissues, IFP range from −8 to +6 mmHg	Increased IFP (can reach 50 or even 100 mmHg)
Tissue‐level Biochemical factors		
Oxygen level	Normoxia	Hypoxia, dysregulated angiogenesis
pH	Extrace llular pH ~7.4	Extracellular pH (6.5–6.9)
Metabolism	Normal metabolism (glucose converted to pyruvate) Normal mitochondrial function Low lipid oxidation, high antioxidant enzymes, Low ROS (low resistance of cells to ROS)	Metabolic reprogramming (enhanced glycolysis and production of lactate), lack of nutrition High lipid oxidation, reduced antioxidant enzymes, enhanced ROS production (cancer cells are resistant to ROS)
Inflammation	Normal inflammation control	Sustained and prolonged inflammation

### ECM remodeling and growth factors

Tumor matrix stiffness significantly influences the biochemical facets of the tumor microenvironment, including the expression, secretion, and activity of growth factors and cytokines. Stiffer matrices can enhance the activation of latent growth factors. For example, TGF‐β, often secreted in an inactive form, is activated by integrin‐mediated interactions influenced by matrix stiffness [40]. In addition, stiff ECM promotes a hypoxic microenvironment, which in turn stimulates the production of growth factors such as VEGF and cytokines that promote angiogenesis and tumor growth [41]. The ECM is vital for intercellular communication, functioning as a reservoir for the sequestration of molecules and as a substrate that facilitates cell adhesion and migration. During the process of cancer invasion and metastasis, the activity of ECM‐degrading enzymes increases, which promotes the release of various matrix‐associated growth factors such as EGF, FGF, HGF, PDGF, and cytokines such as IL‐6, IL‐10, TNF‐α, IL‐1β, and SDF‐1, thereby creating local gradients of released mediators that further promote cancer cell survival, proliferation, and immune evasion [40, 42].

## 3D Cancer models

3D *in vitro* cancer models are biomimetic and can replicate *in vivo* tumors by emulating the complexity of cancer and the TME. It is critical, therefore, to define the purpose of any 3D tissue/cancer model and identify key biomimetic variables. Numerous design parameters need to be considered when engineering 3D cancer models, including the selection of cellular components (cancer/stromal cells), incorporation of biomimetic ECM, replication of fluid flow, establishment of biochemical gradients, and the development of either a systemic multi‐organ model for studying metastasis or isolated single organ models [43].

Patient‐derived cancer cells and immortalized human cancer cell lines are commonly used cell sources. While standardized, robust immortalized cancer cell lines are mostly used for the development of 3D models, they only embody one of the numerous phenotypes found in the primary tumor and typically become less physiologically relevant with continuous passaging. Patient‐derived cancer cells recapitulate tumor heterogeneity, but culturing these cells is challenging as the maintenance of the relevant genotype/phenotype is difficult [12]. Inclusion of tumor‐specific essential stromal cell types, biomimicry of the microenvironmental ECM composition and architecture, as well as biomimetic biomechanical stimuli is vital to replicate the spatial organization of tissues. The assembly of complex 3D cell structures can facilitate the recapitulation of relevant biochemical cues [43]. Such 3D tumor models that showcase tumor/stromal cross‐talk can be classified into organoids, engineered tissue models, *ex vivo* models, bio‐printed, and organ‐on‐a‐chip (OOC) models.

### 3D models used to test interdependence of multiple facets of the TME

#### Biophysical‐stromal cell interactions

All cells within the TME experience different mechanical forces, including compressive, tensile, and shear forces. These biophysical facets influence and direct cancer progression (Fig. 1). It is important to explore how each cell type responds to these mechanical stimuli and how interactions between forces and cells collectively influence tumor progression. Mechanical cues influence the interaction between cancer cells and various immune cell types, such as T and B cells, macrophages, natural killer cells, and dendritic cells, which depend on physical interactions with one another to activate their responses. Co‐culture of MDA‐MB‐231 breast cancer cells with monocytes and endothelial cells in the presence of interstitial fluid flow significantly enhances the activation of monocytes to tumor‐associated macrophages (TAM)‐like phenotype through stimulation by colony‐stimulating factor 1 (CSF‐1) [44]. The resultant activated macrophages, in conjunction with interstitial fluid flow, subsequently facilitate vascular sprouting via the vascular endothelial growth factor (VEGFα) signaling, thereby promoting cancer invasion [44]. Mimicking the desmoplastic matrix of pancreatic ductal adenocarcinoma (PDAC) through the fabrication of a 3D tumor niche using dual‐crosslinking gelatine methacrylate/hyaluronic acid methacrylate hydrogels generates an immunosuppressive microenvironment, marked by a decrease in M1 markers and an increase in M2 markers in TAMs, induced by the PI3K‐AKT‐SELE/VCAM1 axis [45]. Increased matrix density and alignment of collagen fibrils result in reduced T‐cell proliferation, suppressed T‐cell activation, and altered migratory behavior of T cells, which impedes their ability to infiltrate tumor environments, mainly mediated by enhanced YAP signaling [46]. Cancer cells are enveloped by a dense layer of glycosylated proteins and lipids, known as the glycocalyx, which plays a vital role in fostering a favorable tumor immune microenvironment (TIME) [47]. Nearly all cancers display modifications in glycan synthesis, resulting in dysregulated levels of glycans which may possess modified structural properties, profoundly impacting cancer progression. It is suggested that a dense glycocalyx acts as a protective barrier, preventing tumor cell ligands from interacting with the receptors of immune cells [48]. Using a 3D synthetic bone matrix model, upregulation of glycosylation of breast cancer cells was found to increase the thickness of the cancer cell glycocalyx, leading to evasion of the cytotoxic effect of natural killer cells [49].

Replicating the ECM of clear cell renal cell carcinoma in a fibrin‐based 3D culture system highlights the impact of ECM composition and physical properties on the growth of CAF populations [50]. The ECM confers mechanical stability to tissues and cells, allowing for effective cell‐matrix interactions that govern tissue function. Furthermore, the ECM harbors several types of growth factors that support cell adhesion, growth, and migration [51]. Although often overlooked in cancer research, the density and composition of the ECM are recognized as crucial for the progression of cancer. Laminin is a significant element in the modulation of endothelial cell (EC) morphology and the formation of vascular networks, associated with increased tumor invasion into the stroma of engineered compartmentalized biomimetic colorectal tumouroids [52]. Laminin has been previously shown to increase the expression of VEGFR2 receptors by ECs leading to enhanced VEGF uptake and resulting in the development of end‐to‐end networks in 3D hydrogels [53].

#### Biophysical‐biochemical interactions

The biophysical microenvironment of the TME, which includes the increased stiffness or fibrosis associated with tumors, can impact the biochemical microenvironment in a multitude of ways (Fig. 1). Matrix remodeling can alter the diffusion of gradients of cytokines and growth factors, as well as alter growth factor sequestration. There have been a multitude of studies in 3D tumor models that have helped decipher the relationship between these TME facets.

PDAC is an aggressive solid tumor, distinguished by a dense fibrotic stroma that significantly contributes to its aggressive nature. This dense stroma leads to elevated interstitial pressures and hypoxia development, which subsequently creates a nutrient‐deficient environment that necessitates a modification of metabolic requirements. A microfluidic PDAC‐on‐a‐chip platform encompassing pancreatic cancer cells and pancreatic stellate cells (PSCs) embedded in a 3D collagen matrix enabled the correlation of matrix stiffness and solid stress to the altered metabolism of the PDAC TME observed clinically in patients with pancreatic cancer [54].

The mutual interaction between ECM remodeling and hypoxia signaling substantially impacts tumor progression and metastasis. The physical characteristics of the ECM, including the diameter and alignment of fibers, pore size, and viscoelasticity, modulate hypoxia‐driven biochemical processes, which in turn facilitate metastatic transformation [55]. Mechanical forces induced by a stiff matrix are transmitted via mechanosensitive focal adhesion proteins to biochemical signaling in the cells [56]. Several matrix stiffness‐sensitive transcription factors are involved in cancer progression, such as YAP/TAZ, NF‐κB, Snail, and HIF1A [56]. Culturing glioma cells in a stiff ECM enhances HIF1A expression, which subsequently increases the expression of tenascin C, a critical component in the aggressiveness of gliomas [57]. Additionally, in breast cancer patients, there is a notable positive correlation between the expression of HIF1A and the stiffness of the affected tissues [58]. Reciprocally, hypoxia enhances ECM remodeling by stimulating protein degradation, modifications in composition, and changes in structural organization, ultimately resulting in a fibrotic/stiff ECM [59]. Under hypoxic conditions, an increase in the secretion of MMPs facilitates the degradation of the basement membrane ECM through HIF signaling pathways [60]. A hypoxic environment drives augmented deposition and crosslinking of collagen, fibronectin, and hyaluronic acid [61]. Furthermore, it has been suggested recently that hypoxia and the ECM collaborate to modify various aspects of cellular metabolism [62]. This partnership facilitates the enhancement of aerobic glycolysis through the upregulation of glucose transport mechanisms and glycolytic enzymes, in addition to the modulation of intracellular pH levels. Likewise, both components influence lipid and amino acid metabolism by promoting the uptake and synthesis of these macromolecules, thus supplying the tumor with supplementary energy crucial for growth and metastatic progression. The incorporation of breast cancer cells in synthesized collagen matrix scaffolds with controlled porosity and tortuosity facilitates the development of a hypoxic environment impacting cancer cell growth dynamics and the promotion of aggressive phenotypes [63]. Another study reported the successful recapitulation of a hypoxic TME in a 3D‐printed *in vitro* model of patient‐derived PDAC cells embedded in a biomimetic ECM, resulting in markedly enhanced glutamine catabolism, further exacerbating hypoxia in tumors [64].

#### Stromal cell‐immune cell interactions

Cell–cell interactions play a crucial role in cancer progression, and deciphering these precise interactions is difficult given the number of different cell types at play. Even within animal models, these challenges persist. 3D tumor models provide an opportunity to engineer biomimetic scenarios to specifically understand cell–cell interactions in a systematic and controlled manner (Fig. 1). These tools are powerful in helping unravel cell–cell communication.

Colorectal cancer (CRC) evolves within a multifaceted TME, characterized by the dynamic interactions between cancer cells and various types of stromal cells. In advanced stages of CRC, the stroma can constitute as much as 50% of the primary tumor mass, predominantly consisting of mesenchymal stromal cells (MSCs). Mesenchymal cell signatures, in conjunction with tumor‐promoting macrophages, are significantly linked with disease progression and diminished overall prognosis in CRC [65]. Incorporation of MSCs, monocytes, and CRC cells in a gelatine/methacryloyl‐based hydrogel demonstrated enhanced expression of matrix remodeling proteins FN1 and MMP9, induced release of tumor‐promoting immune molecules MIF, Serpin E1, CXCL1, IL‐8, and CXCL12 by MSCs, leading to the suppression of the anti‐tumor functions in macrophages [66]. Triculture of pancreatic cancer cells, PSCs, and ECs in 3D leads to inhibition of the T‐cell inflammatory response mediated through several mechanisms, including secretion of suppressive soluble factors (IL‐6, SDF‐1, galectin‐1), excess deposition of ECM to limit T‐cell infiltration, and metabolic reprogramming that depletes nutrients causing T‐cell exhaustion [67]. Studies using co‐culture models of hepatocellular carcinoma (HCC) and ECs reveal that endothelial cells trigger an inflammatory response in HCC cells, leading to an upregulation of the TNF signaling pathway. Furthermore, the interaction between HCC and endothelial cells facilitates macrophage polarization towards a pro‐inflammatory and pro‐angiogenic phenotype [68]. Pro‐inflammatory cytokines and growth factors produced by immune cells can induce the transformation of fibroblasts into myofibroblasts, which are characterized by an increased expression of contractile protein genes, such as alpha smooth muscle actin (Acta2), as well as other ECM proteins. Consequently, these diverse fibroblast populations can modify the physical characteristics of the ECM. Sophisticated organoid TME models, encompassing tumor, stromal, and immune components, result in the activation of myofibroblast‐like CAFs and the infiltration of lymphocytes in the tumor [69].

#### Stromal cell‐biochemical interactions

Gradients of chemokines, cytokines, growth factors, small molecules like oxygen, and pH act synergistically to promote the directional movement of tumor cells towards the vasculature and lymphatic systems [70]. A 3D bio‐printed model revealed that the interaction of co‐cultures of two glioblastoma cell lines (U‐251 and DK‐MG) with mesenchymal stromal cells resulted in a significant rise in the repertoire and concentrations of secreted chemokines compared to monocultures [71]. Another study used 3D *in vitro* PDAC tumor models to examine the interactions of macrophages and cancer cells in hypoxic settings in the presence of various chemical and molecular gradients and demonstrated that hypoxic macrophages reprogrammed tumor cell responses, evidenced by higher TGF‐β and PD‐L1 expression compared to monocultures of tumor cells [72]. A different study demonstrated that an acidic TME stimulates the NF‐kB‐mediated inflammatory response of the tumor‐associated mesenchymal stromal cell through increased secretion of several cytokines and chemokines, including IL‐6, that further promote cancer invasiveness in a 3D microfluidic model of osteosarcoma [73]. Tumor‐associated environmental factors such as lack of nutrients, buildup of waste products, and acidic pH hinder the antitumor functions of T and natural killer (NK) cells. Tumor‐on‐a‐chip models encompassing either MCF‐7 or patient‐derived breast cancer cells and endothelial cells replicate gradients of nutrients and pH, resulting in biomimetic cell proliferation and necrosis characteristic of solid tumors [74]. These models further demonstrate the gradual reduction of the cytotoxic potential of NK cells leading to their exhaustion [74]. Notably, even after alleviating the environmental stress imposed by the tumor, NK cells did not revert to a non‐exhausted state, exhibiting various molecular and functional changes [74].

### 3D models for studying how tumor‐stromal interactions impact drug resistance

Drug resistance poses a significant challenge in the treatment of cancer. Despite continuous developments of new compounds and drug combinations that exhibit greater effectiveness in eradicating cancer cells, the onset of drug resistance is still inevitable [75]. A recent novel concept proposed that tumor resistance to antineoplastic agents may arise not only from cell‐autonomous mechanisms that involve genetic and epigenetic alterations in tumor cells, but also from non‐cell‐autonomous mechanisms that are related to the TME [76]. While the majority of cell‐autonomous mechanisms have been validated in preclinical models and in clinical settings, preclinical *in vitro* models that extensively examine TME‐related mechanisms of drug resistance are still limited [76]. Thus, a comprehensive understanding of these complex interactions through 3D *in vitro* models is critical for the development of effective therapeutic strategies. A multi‐compartmentalized vascularized tumor‐on‐a‐chip model of ovarian cancer demonstrated the role of CAF‐mediated ECM remodeling and formation of vessel‐like structures with associated hypoxia gradients in the promotion of drug resistance to carboplatin/paclitaxel treatment, henceforth reversed by targeting TGF‐β signaling [77]. A 3D breast tumor model [78] validated the role of the PAK1 pathway in CAF differentiation, migration, and contraction, thereby aggravating chemotherapy resistance. This was successfully reversed through fabricated polymeric nanofibers loaded with PAK1 inhibitors, which enhanced paclitaxel efficacy as evidenced by >90% reduction in cancer viability with diminished collagen type I and α‐smooth muscle actin [78]. The interplay between colorectal cancer organoids and CAFs in organoids influences the expression of genes associated with 5‐fluorouracil and oxaliplatin resistance, particularly in pathways linked to interferon‐alpha/beta signaling and the assembly of major histocompatibility complex class II protein complexes measured by transcriptome profiling following anticancer drug treatment [79]. A similar study reported the development of resistance of cancer cells to gemcitabine, 5‐FU, and paclitaxel in co‐cultures of patient‐matched PDAC organoids and CAFs. Resistance was mediated by the induction of a pro‐inflammatory phenotype in CAFs associated with increased expression of EMT‐related genes [80]. A TME on‐chip comprising breast cancer cells and ECs cultivated under controlled interstitial pressure conditions demonstrated that cancer cells exposed to doxorubicin showed greater survival rates than those in a 2D culture environment, highlighting the influence of elevated IFP on drug delivery into tumor tissues [81].

Cancer cells and mesenchymal stem cells/adipose‐derived stem cells (MSCs/ASCs) can interact through several mechanisms, including tunneling nanotubes (TNTs), cell–cell fusion, and the transport of extracellular vehicles (EVs) [82]. These mechanisms enable the exchange of diverse intracellular components, including macromolecules, organelles, vesicles, proteins, calcium ions, and other substances. Patient‐derived breast cancer organoids cultured with ASCs showed mitochondrial transfer via TNTs, which was associated with triggered metabolic alterations and enhanced ATP production that boosts the efflux activity of ABC transporters and promotes multidrug resistance (MDR) [83]. Tumor metastasis is a major cause of cancer‐related deaths, as tumor cells spread from the original location to other areas such as the liver, bone, brain, lung, and lymph nodes [84]. This process involves three main stages: detachment of tumor cells from their original site, migration through the bloodstream to a secondary location, and adjustment to survive in new sites by forming micro‐metastases. The process of organ‐specific metastasis involves the complex interplay of cancer cells, the microenvironment of each organ, the distinct attributes of the vascular network in each organ, and the immune system [85]. Breast‐derived tumor cells migrate through the vascular system, infiltrating perivascular niches located around blood capillaries. The slow blood flow maintains these niches by transporting oxygen, nutrients, and signaling molecules from the bloodstream into the surrounding interstitial tissue [86]. Additionally, the ECM, ECs, and mesenchymal stem cells play a crucial role in regulating the homing of metastatic cells. A perfused bone perivascular niche‐on‐a‐chip was designed to explore breast cancer progression and drug resistance in bone colonization [87]. Delivering controlled flow velocities, shear stresses, and oxygen gradients to the niche‐on‐a‐chip facilitated the establishment of a long‐lasting, self‐assembled vascular network in this model. Breast cancer cells exposed to interstitial flow within the bone perivascular niche‐on‐a‐chip exhibit a slow‐proliferative state that correlates with increased sunitinib resistance [87].

Brain metastasis in non‐small cell lung carcinoma (NSCLC) is fatal and is known for its resistance to cancer therapies, which could be attributed to the tumor microenvironment. Tumor cells from brain metastatic NSCLC (BM‐NSCLC) engage in dynamic interactions with the brain TME (bTME) which plays a crucial role in forming a brain metastatic niche [88]. A 3D microfluidic system established the reciprocal communication between BM‐NSCLC, astrocytes, and brain‐specific ECs. This tumor‐stroma interaction resulted in the activation of NF‐κB, MAPK, and Notch signaling pathways, immune and inflammatory responses associated with increased resistance of bTME+ BM‐NSCLC to afatinib compared to monocultures [89].

## Methods of analysis of tumor‐stroma interaction in 3D models

The complexity of 3D cancer models presents challenges in data collection and analysis due to inherent heterogeneity and interference from the matrix. Developing strategies for distinguishing between different cell populations in the TME is of utmost necessity. Additionally, tools for evaluating drug responses in 3D cultures hold great potential for advancing drug discovery [90]. Analysis of 3D culture models will greatly depend on the research question and the intended application of this model. These models serve two primary purposes, which are the mechanistic study of cancer biology and the development of novel and personalized therapies. Studies examining tumor cells often assess the 3D engineered cancer mass as a whole to ascertain growth, spheroid size, invasion, metastasis, and cancer cell proliferation/apoptosis. There are a wide range of assays and microscopy techniques that are able to assess these parameters.

Studying the TME usually includes investigating angiogenesis, immune cell infiltration, and inflammation, ECM dysregulation, heterogeneous stromal cell function, a hypoxic/acidic microenvironment, and metabolic alterations. These require analysis methods that are indirect and often require multiple modes of assessment. For example, angiogenesis as a process may include studying an increase in angiogenic growth factors at the gene or protein level, along with measuring the complexity of cell‐to‐cell network aggregation by endothelial cells. Collectively, these data will provide an understanding of changes or remodeling in vascularization.

**Fig. 2:**
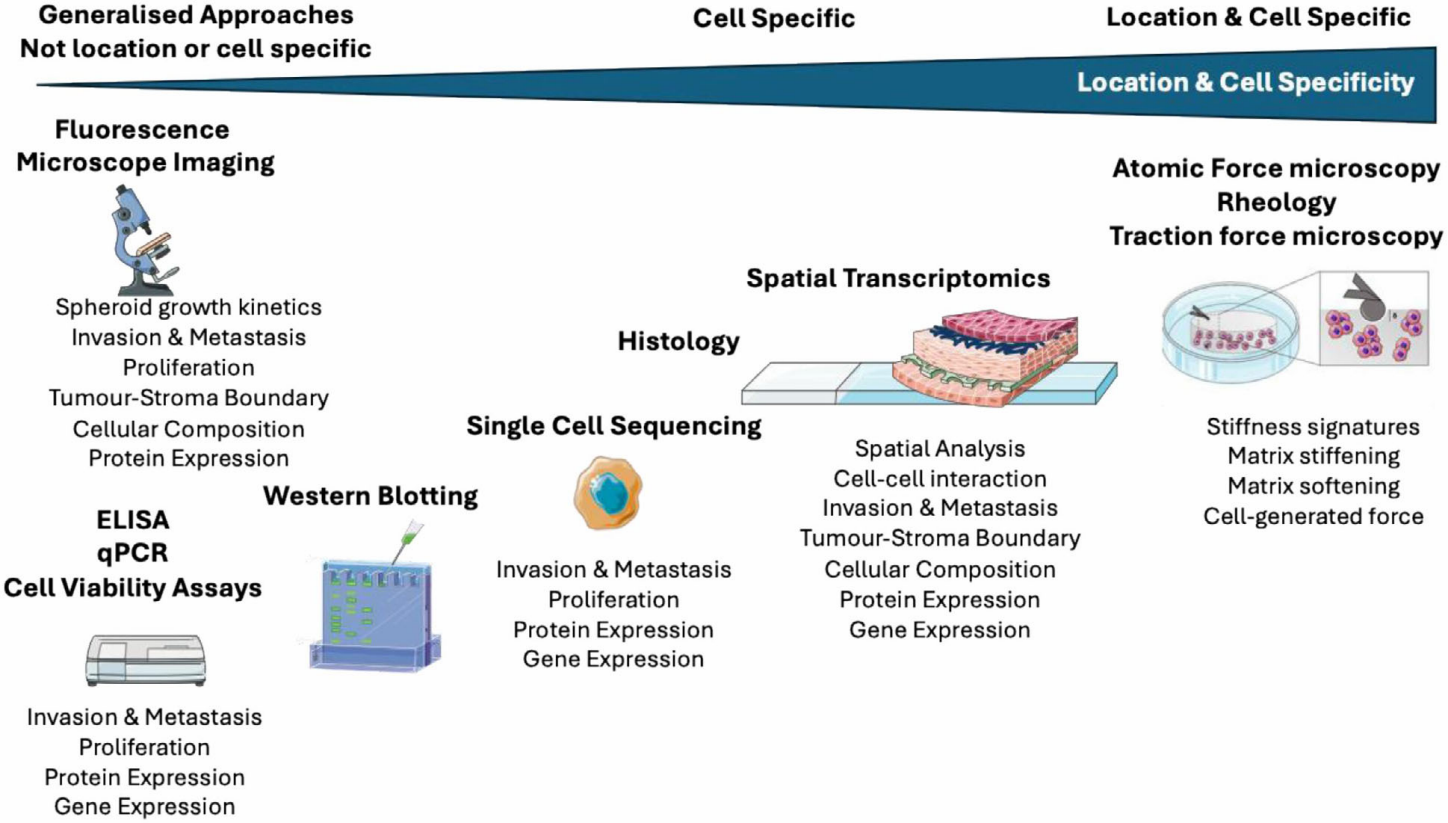
Schematic of the multiple approaches taken to analyze 3D models. We start with general techniques and then show methods that provide details on spatial analysis. Specialized techniques can be modified to provide analysis for 3D tumor models, including the use of atomic force microscopy to measure tissue/matrix remodeling by cancer cells. Schematic generated using Servier Medical Art.

The disruption of signaling mechanisms which lead to increased growth and survival of tumors can stem from various sources such as changes in the DNA sequence, the epigenetic landscape, the levels of gene and protein expression, and the functional activity of proteins, including their interactions and post‐translational modifications. Thus, comprehensive understanding requires analysis at different levels within the same cellular context [91]. Tumor‐stroma interactions in 3D models can be analyzed using a variety of techniques (Fig. 2).

Traditional techniques such as histology and quantitative PCR have long been the gold standard to correlate proteins and genes with disease phenotype. These methods are still used in pathology and often rely on clinicians and pathologists using the accrued knowledge of marker regulation with disease progression and/or regression. This means the compatibility of 3D models with existing techniques is critical for comparisons to human tissue. An example is the fixing and sectioning of 3D models, which allows for direct comparison of disease progression [91, 92]. This dataset also provides the basis for the underlying validation of any 3D model as a biomimetic equivalent of diseased human tissue.

There are technologies now being employed to bring together large sets of data to gain further insight or details into disease (Fig. 2). This includes the use of spatial transcriptomics to study patterns of gene regulation in spatially relevant locations, for instance, at the tumor‐stroma boundary versus deep within the tumor core [93]. Furthermore, atomic force microscopy has allowed for the study of cell‐generated matrix remodeling to be studied [94, 95]. Employing more sophisticated techniques will give us greater insight into disease progression and allow us to study multiple facets of therapeutic intervention.

## Conclusions and future directions

To summarize, cancer is a multifaceted disease where the interactions between tumor cells and the different components of their surrounding microenvironment impact tumor progression and therapeutic efficacy. The development of biomimetic *in vitro* systems of the TME is essential for cancer modeling to facilitate the development of novel therapeutic strategies and decipher the molecular processes underlying tumorigenesis. The evolution of 3D *in vitro* tumor models that offer controlled and physiologically relevant conditions enables researchers to isolate the effects of both cellular and acellular microenvironmental factors on cancer progression. Despite their advantages, 3D models are still constrained by inherent drawbacks that might limit their translation into clinical settings. These include incomplete recapitulation of heterogeneous subpopulations of cancer cells, the discrepancy between the biology of the model system and the context of the human body, the design of the 3D structure, like cellular composition and biomaterial formulation, and the quantification of the 3D model. In the future, the application of machine learning to replicate and predict the behavior and properties of 3D structures according to their cellular and material composition could facilitate the rapid advancement of personalized tissue model development. This could be accomplished through the systematic selection of cellular components and biomaterials and meticulous control of environmental conditions. The integration of patient‐derived tissues, cutting‐edge imaging, multi‐omics technologies, and computational models has substantial potential to revolutionize the field of personalized medicine, particularly in oncology.

## Conflict of interest

The authors confirm that they have no conflicts of interest to disclose.

## Author contributions

STR wrote the main part of the original draft and prepared the tables. DB generated the figures and helped edit the draft. UC and AJM were involved in the concept development and edited the review. All authors have read and agreed to the published version of the manuscript.
